# Genetic Diversity and Conservation Priority of Korean Chicken Breeds Using Single-Nucleotide Polymorphism Markers

**DOI:** 10.3390/ani15081084

**Published:** 2025-04-09

**Authors:** Huimang Song, Yoonsik Kim, Seunghwan Lee, Bonghwan Choi, Seungchang Kim, Daehyeok Jin, Gaeun Kim, Seungmin Ha, Seongsil Mun, Youngchul Cho, Yoonji Chung

**Affiliations:** 1 Animal Genetic Resources Research Center, National Institute of Animal Science, Rural Development Administration, Hamyang 50000, Republic of Korea; hope0909@korea.kr (H.S.); bhchoi@korea.kr (B.C.); kisc@korea.kr (S.K.); jdh1662@korea.kr (D.J.); gaeun2010@korea.kr (G.K.); justusha@korea.kr (S.H.); mss1021@naver.com (S.M.); lily1961@korea.kr (Y.C.); 2 Institute of Agricultural Science, Chungnam National University, Daejeon 34134, Republic of Korea; watogo@hanmail.net; 3 Division of Animal & Dairy Science, Chungnam National University, Daejeon 34134, Republic of Korea; slee46@cnu.ac.kr

**Keywords:** Korean native chicken, Korean locally adapted chicken, genomic characterisation

## Abstract

Maintaining genetic diversity is vital for biodiversity and livestock sustainability, with monitoring serving as a prerequisite for conservation strategies. As an initial step for developing future strategies, we analysed the genetic diversity and conservation priorities of 16 chicken breeds managed by a government institute in this study. The breeds were classified as native or locally adapted per FAO definitions and grouped by origin (i.e., governmental or private farms). Genetic diversity, assessed through observed and expected heterozygosity, inbreeding coefficients, and linkage disequilibrium, was high in native breeds from government institutions, such as Chungnam White Korean Native (CNW) and Gangwon Black Korean Native (GWL). Private farm breeds, including Hoeungseong-Yakdak (HYD), exhibited lower diversity. Conservation priorities based on contributions to total genetic diversity (HT) and allelic diversity (AT) revealed that locally adapted breeds, such as Cornish (S) and White Leghorn, significantly contributed to HT. Native breeds, such as CNW and Hyuin black, had higher contributions to AT. Combined assessments indicated that S, CNW, and GWL have the highest conservation priorities. Despite lower rankings, unique breeds such as HYD require targeted conservation owing to their distinctiveness. In conclusion, Korean chicken breeds such as S, CNW, and GWL are vital for maintaining genetic diversity among government-managed breeds.

## Simple Summary

Genetic diversity is important for biodiversity and sustainable livestock development, and monitoring is an essential preliminary analysis that must be conducted before establishing conservation strategies. This study evaluated 16 chicken breeds in Korea that were categorised into native and locally adapted breeds based on their origin from either government-managed or private farms. Genetic diversity, assessed through variation within populations and inbreeding levels, was high in native breeds from government farms, while those from private farms exhibited lower diversity, increasing their vulnerability. Locally adapted breeds contributed significantly to the total genetic diversity, highlighting their role in maintaining overall genetic variation, while native breeds contributed strongly to allelic diversity, preserving unique genetic traits. Combining these metrics identified breeds, such as Chungnam White Korean Native, Gangwon Black Korean Native, and Cornish, as the highest priorities for conservation. The study findings offer a focused approach to preserving genetic resources and ensuring sustainable livestock populations.

## 1. Introduction

Genetic diversity is essential for sustaining biodiversity and effective conservation [1]. In livestock, preserving genetic diversity is vital for the sustainability of industry, as it enhances animals’ resilience to diseases, environmental changes, and market fluctuations [2,3,4]. Chickens, in particular, play an important role in genetic diversity among domestic animals; they are a specialised area in scientific research and serve as a primary food source owing to their extensive genetic variation and adaptability to various environments globally [5,6]. Native chicken breeds exhibit diverse phenotypes, such as unique comb shapes, feather colours, and shank colours [7]. These phenotypic differences reflect a high level of genetic diversity, which is critical for maintaining a broad gene pool and preventing the loss of genetic resources from ancestral lines [8]. Despite the importance of genetic diversity and the value of chickens, conservation efforts within the domestic chicken industry are insufficient, particularly regarding native breeds. This oversight poses a significant risk to maintaining the genetic diversity necessary for the long-term sustainability of the poultry industry [9,10].

In Korea, the government has implemented legal and administrative measures to protect agricultural bio-resources. Notably, in 2007, following the first International Technical Conference on Animal Genetic Resources organised by the United Nations Food and Agriculture Organization (FAO) in Interlaken, the Korean government enacted the Act on the Conservation, Management, and Use of Agricultural Bio-Resources [11,12]. This legislation led to the allocation of a dedicated budget to conserve genetic diversity in native and locally adapted livestock breeds through two approaches: ex situ, by establishing conservation facilities, and in situ, by providing financial support to local governments and private farms. The Animal Genetic Resources Research Center (AGRRC) of the National Institute of Animal Science is responsible for conserving domestic animal breeds [12]. Among the various chicken breeds in Korea, the AGRRC has conserved 43 endangered native chicken breeds and strains through both in situ and ex situ conservation efforts, excluding those currently preserved through cryopreservation. While these breeds and strains are monitored by the government, there remains a lack of targeted conservation programmes tailored to the genetic and population status of individual breeds and strains. To address this, it is necessary to employ advanced technologies to systematically evaluate the genetic and population status of these breeds. Furthermore, establishing an effective conservation framework requires prioritising breeds and strains through a comprehensive assessment of their genetic characteristics and conservation importance.

Genetic characterisation should first be performed to inform the decision-making process for conservation programmes aimed at preserving the genetic diversity of farm animal populations [13]. In recent years, molecular technology has been extensively applied to assess the genetic diversity of chicken populations. Among the major markers used to measure genetic diversity, microsatellite markers and single-nucleotide polymorphism (SNP) have been the most commonly employed. SNPs have become more highly favoured than microsatellite markers recently owing to the high level of variation both within and between chicken breeds [1,13,14]. Genetic diversity and conservation prioritisation based on SNP markers have gained significant momentum in recent years. For instance, a genome-wide SNP analysis of 23 Italian chicken breeds identified clear genetic differentiation among breeds, revealing distinct population structures. This study emphasises the importance of monitoring genetic variability and designing effective conservation plans tailored to the specific needs of breeds [15]. In the context of indigenous chickens, genome-wide analyses unveiled high genetic diversity within populations, such as the study on South African village chickens. This study underscored the unique genetic components retained in local breeds, which are essential for biodiversity and long-term sustainability [16]. Moreover, several previous studies have evaluated the conservation priority of chicken breeds through genetic characterisation, using the contribution of each breed to overall genetic diversity [17,18].

To the best of our knowledge, no previous studies have evaluated the conservation priorities of Korean native chicken breeds based on their contribution to overall genetic diversity. In this study, we aimed to assess the genetic diversity and population structure of Korean native chicken breeds and strains, including 12 native breeds and 4 locally adapted breeds. We used the FAO standard to define native and locally adapted breeds in this study [19]. Locally adapted breeds are defined as breeds originally established in other countries that have been clearly recorded as being reared in the country for over 40 years or across more than six generations [19]. To avoid redundancy, the breeds and strains were selected based on phenotypic traits, and only 16 non-duplicated breeds were chosen from the 43 breeds conserved through in situ and ex situ efforts. Additionally, we identified the conservation priorities of Korean native chickens by employing methods that assess their contribution to overall genetic diversity.

## 2. Materials and Methods

### 2.1. Sample Collection and Genotypes

This study was approved by the Institutional Animal Care and Use Committee of the National Institute of Animal Science, Republic of Korea (approval number: NIAS-2023032). The experimental animals were not anaesthetised or euthanised in this study. All methods for the animal experiments are reported in accordance with the ARRIVE guidelines (https://arriveguidelines.org (accessed on 8 April 2025)).

Based on the phenotypic traits from 43 breeds conserved through in situ and ex situ efforts, 16 non-duplicated breeds were used in this study (Table 1). These breed populations were managed separately for sufficient generations; thus, potential sampling bias was not considered. A total of 315 samples comprising purebred Korean chicken breeds, including native and locally adapted breeds, were analysed in this study in 2023. The blood samples of Hoeungseong-Yakdak (HYD) and long-tail chicken (LTC) were collected from private farms. Blood samples from the other breeds were obtained from the AGRRC, as these breeds are conserved ex situ at the AGRRC farm. Genomic DNA (gDNA) was extracted from blood samples from all chickens using a genomic DNA purification kit (Promega, Madison, WI, USA). The concentration and purity of extracted gDNA were investigated using a NanoDrop spectrophotometer (Thermo Fisher Scientific, Waltham, MA, USA). The gDNA was stored at −20 °C until use. With these gDNA samples, data regarding 57,636 SNPs were obtained using an Illumina chicken SNP 55K bead chip (Illumina, San Diego, CA, USA).

### 2.2. Data Filtering

Initial quality control of the genotype data was performed using PLINK v1.9 [20]. SNPs were filtered to retain those with a missing genotype rate <10% and a minor allele frequency (MAF) >5%. SNPs located on sex chromosomes (n = 6270) were excluded to focus the analysis on the 28 autosomes. After filtering, 47,044 high-quality SNPs remained for subsequent analyses.

### 2.3. Population Structure and Genetic Diversity

Principal component analysis (PCA) was conducted using PLINK v1.9 to investigate the genetic structure among the chicken populations. Inbreeding coefficients ($F_{HOM}$) for each breed were also calculated using PLINK v1.9. A phylogenetic tree based on Nei’s genetic distance was constructed with the ape package v5.8 in R [21], providing insights into the genetic relationships among the populations. Genetic diversity within and between populations was assessed using several metrics calculated with the Metapop2 software v1.0.0, which effectively models genetic drift and migration patterns for population structure analysis [22]. Nei’s minimum genetic distance (DNei) was calculated using the following formula:

$$DNei=\frac{J_{X}+J_{Y}}{2-J_{XY}}$$

where $J_{X}$ and $J_{Y}$ represent the probabilities that two randomly chosen alleles from populations X and Y, respectively, are identical, and $J_{XY}$ is the probability that one allele from X and one allele from Y are identical [23]. The pairwise population differentiation index (Fst) was calculated according to Weir and Cockerham (1984) [24].

$$Fst=\frac{\sigma^{2}}{\bar{p}(1-\bar{p})}$$

where $\sigma^{2}\;$ is the variance of allele frequencies among subpopulations, and $\bar{p}\;$ is the average allele frequency in the total population. Total genetic diversity (HT) was partitioned into within-population diversity (HS) and genetic divergence between populations (DG) using the following equation:

$$HT=HS+DG=\left(1-\bar{f}\right)+DG$$

where $\bar{f}$ is the average coancestry within subpopulations [22,25,26]. Total allelic diversity (AT) was also partitioned into mean allelic richness across all subpopulations (AS), and the average number of unique alleles between subpopulations was calculated as follows (DA):

AT=AS+DA

Here, $AS=\frac{1}{n}\sum_{i=1}^{n}k_{i}$, where $k_{i}$ is the number of alleles in a subpopulation, i, and n is the total number of subpopulations. The equation $DA=\frac{1}{n(n-1)}\sum_{i\neq j}\left(k_{i}-k_{ij}\right)\;$ represents the average number of alleles unique to a subpopulation when compared to others, with $k_{ij}$ being the number of shared alleles between subpopulations i and j.

The indices of within-breed diversity, including average coancestry between individuals ($f_{ii}$), the average self-coancestry of individuals ($s_{i}$) [27], observed heterozygosity ($H_{O}$), and expected heterozygosity ($H_{E}$), were calculated to identify priority breeds for conservation. Linkage disequilibrium (LD) decay was analysed using PLINK v1.9, and the results were visualised to assess the extent of LD across the genomes of different populations and within-population genetic diversity and to identify breeds undergoing genetic erosion for conservation prioritisation.

Ancestry proportions of individuals from the 16 groups were estimated using the ADMIXTURE software v.1.3.0 [28], which was selected for its accuracy and efficiency in inferring population structures from SNP data.

## 3. Results

### 3.1. Population Structure and Genetic Diversity Analysis

#### 3.1.1. Population Structure

**Figure 1:**
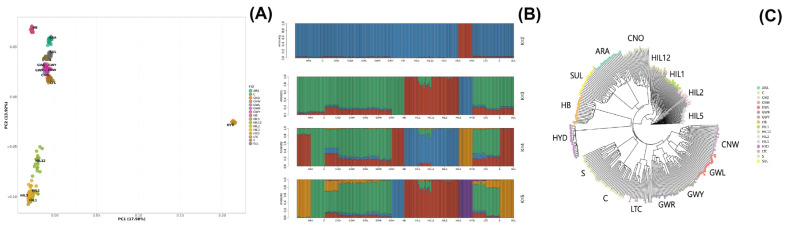
Genetic relationships among the Korean native and locally adapted chicken breeds evident from principal component analysis (**A**), admixture analysis (**B**), and phylogenetic analyses (**C**). In PCA (**A**), the first two principal components (PC1 = 17.98%, PC2 = 13.92%) showed that Korean native breeds (GWL, GWY, GWR, CNW, CNO, LTC) formed a single cluster, indicating high genetic similarity, while HYD and HIL groups were distinct. In admixture analysis (**B**), genetic differentiation was evident, with HYD separated at K = 2 and HIL groups separated at K = 3. At K = 5, the six Korean native breeds clustered together, suggesting a shared genetic background. In the phylogenetic tree (**C**), Nei’s genetic distance confirmed that the six Korean native breeds formed a single cluster, with CNW, GWL, GWR, and GWY showing particularly strong genetic connections, consistent with PCA and admixture results.

After the pre-filtering of SNPs, PCA was performed using 47,044 SNPs to assess the genetic relationships among 16 Korean chicken breed populations (Figure 1A). The two principal components of the plot, PC1 and PC2, accounted for 17.98% and 13.92% of the total genetic variation, respectively. Individuals within each population were grouped together. Along PC1, the HYD breed population was distinctly separated from the other groups, showing significant separation from the main cluster of Korean native chicken breeds. Along PC2, the Hyuin black (HIL1), Hyuin yellow (HIL2), Hyuin grey (HIL5), and Hyuin white (HIL12) breed populations formed a separate cluster, with HIL12 being comparatively more distinct. The Korean native breed population, comprising Gangwon black Korean native (GWL), Gangwon yellowish Korean native (GWY), Gangwon reddish Korean native (GWR), and Chungnam white Korean native (CNW), as well as Chungnam Korean Ogye (CNO) and long-tail chickens (LTCs), exhibited overlapping clusters. The locally adapted breeds—Rhode Island (C), Cornish (S), and White Leghorn (SUL)—also displayed overlapping clusters, with the Hwangbomg-dak (HB) and Gyeongbuk Araucana (ARA) breeds showing separation from this main cluster.

The admixture analysis results aligned with the PCA findings, showing clear genetic differentiation among populations. Cross-validation identified the optimal K-value, with results for K = 2 to K = 5 shown in Figure 1B and Supplementary Figure S1. With two common ancestors (K = 2), the HYD breed population was distinctly separated from other breeds. At K = 3, the HIL groups (HIL1, HIL2, HIL5, HIL12) further differentiated. At K = 4, the ARA, C, HB, and SUL breed populations formed distinct clusters. At the optimal genetic component value, K = 5, the HB population was separated from its previous grouping. The analysis ultimately identified six genetic clusters: ARA-SUL, C, CNO-CNW-GWL-GWR-GWY-LTC-S, HB, HIL1-HIL12-HIL2-HIL5, and HYD.

A phylogenetic tree based on Nei’s genetic distance (Figure 1C) grouped individuals by population, which is consistent with the PCA and admixture results. The HIL populations formed a distinct cluster, with HIL12 further separated. Korean native breeds (CNW, GWL, GWR, and GWY) were clustered together with LTC. The ARA and SUL breed populations were closely related.

#### 3.1.2. Genetic Diversity

The observed heterozygosity (Ho) and expected heterozygosity (He) values ranged from 0.0440 (HYD) to 0.3293 (CNW) and from 0.0388 (HYD) to 0.3078 (CNW), respectively. In most breeds, the Ho and He values were similar, indicating that they are close to the Hardy–Weinberg Equilibrium (HWE). However, some breeds, such as HYD (Ho = 0.0440, He = 0.0388) and HB (Ho = 0.1387, He = 0.1353), displayed low heterozygosity. The inbreeding coefficient based on homozygosity (F_HOM_) values ranged significantly from 0.1223 (SUL) to 0.8524 (HYD), with a mean of approximately 0.2687. The F_HOM_ value was the highest for the HYD breed (0.8524), suggesting high levels of inbreeding. Other breeds, such as SUL (0.1223) and CNW (0.1270), displayed relatively lower inbreeding coefficients. MAF values across breeds ranged from 0.0306 (HYD) to 0.2334 (CNW), with an average of 0.1874. Lower MAF values in breeds such as HYD indicated an abundance of rare alleles, potentially due to selective breeding practices that prioritise certain genetic traits. The higher MAF values in breeds such as CNW and GWR (0.2334 and 0.2247, respectively) indicated the even distribution of the alleles. The Fii values ranged from −0.1264 (HYD) to 0.0230 (LTC), with a mean of approximately −0.0503. The negative Fii values in most breeds, including CNW (−0.0646) and GWR (−0.0590), indicated an excess of heterozygosity, possibly resulting from outbreeding. Only LTC shows a positive Fii value (0.0230), indicating a slight excess of homozygosity within this breed. The Si value indicated the consistency pattern of Fii.

#### 3.1.3. LD and Effective Population Size

**Figure 2:**
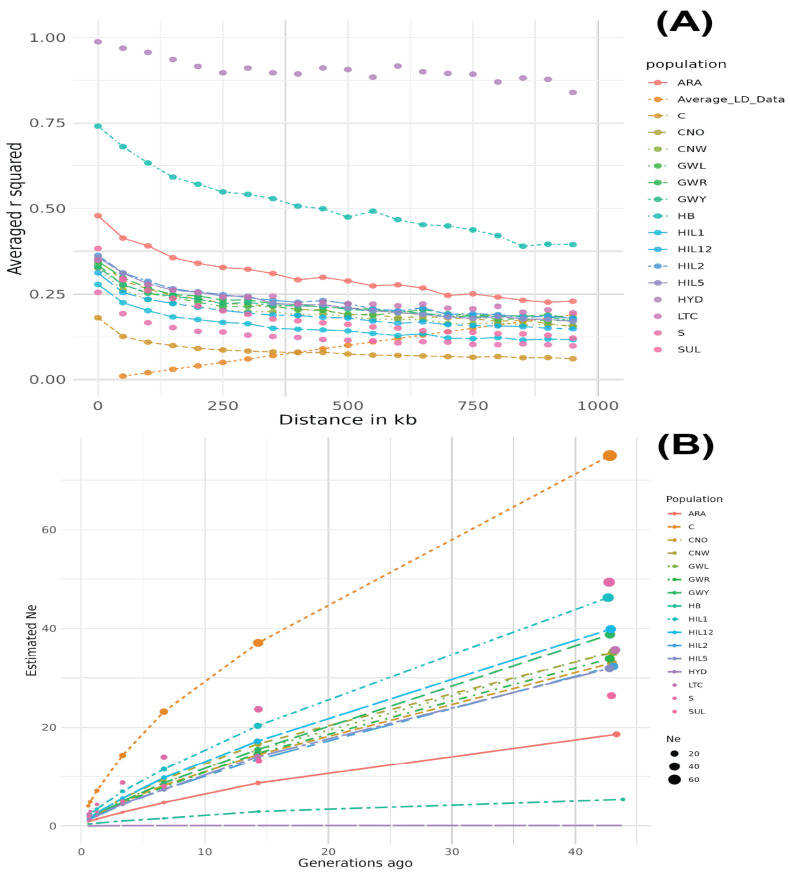
Linkage disequilibrium decay (**A**) and effective population size (**B**) dynamics in Korean chicken breeds. The HYD breed exhibited the highest LD values across all distances, followed by HB and ARA, while C had the lowest LD and highest Ne values.

The HYD breed population exhibited the largest LD values across all distances, with r^2^ = 0.995 at 11.4 kb, which remained high (r^2^ = 0.868), even at 899.2 kb (Figure 2A). The HB breed population showed the second-largest LD values across all distances, although the LD decay was the most pronounced from 0 to 1000 kb compared to other breed populations. The ARA breed population showed the third-largest LD values, while the C breed population exhibited the smallest LD values among all breed populations. The Ne results aligned with the LD decay patterns, with the HYD and HB breed populations showing the lowest Ne values, whereas the C breed had the highest Ne value, which increased dramatically (Figure 2B).

### 3.2. Contribution of Subpopulation to Total Genetic Diversity

#### 3.2.1. Gene Diversity

**Figure 3:**
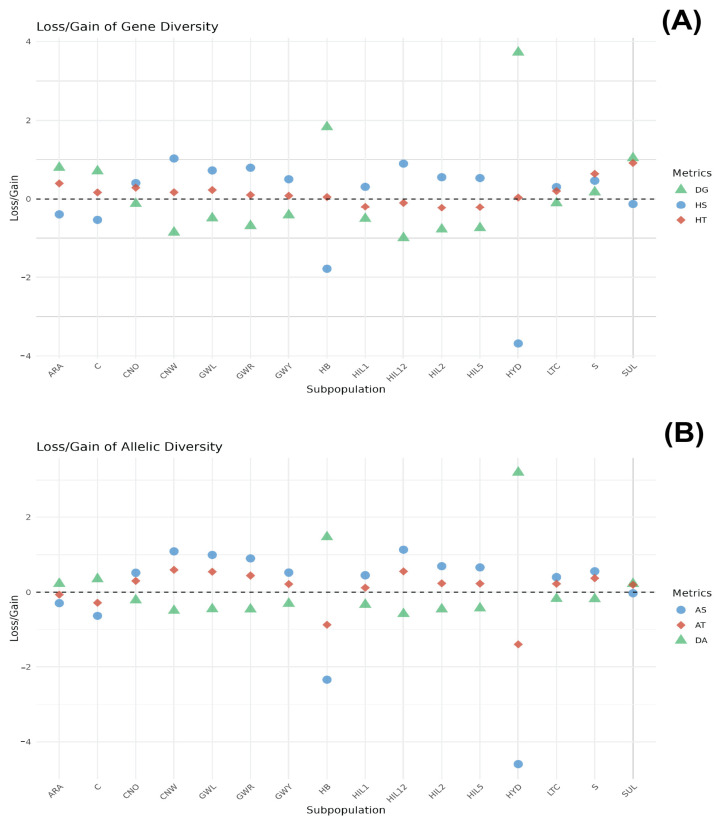
Contribution of each breed within the groups to HT (**A**) and AT (**B**). The SUL breed contributed the most to total gene diversity, while HYD and HB showed the highest DG values with strongly negative HS, indicating their genetic distinctiveness. The ARA breed contributed the most to allelic diversity (AT), while C and SUL exhibited the most negative AT values with highly negative AS and strongly positive DA values, indicating significant inter-breed allelic differentiation.

The genetic contribution of each breed population to total gene diversity (HT) was assessed by partitioning HT into intra-breed (HS) and inter-breed (DG) components, with breeds ranked according to their respective contributions (Figure 3A and Supplementary Table S1). Among the populations, the SUL breed ranked highest in its contribution to HT (0.9169), characterised by a notably negative HS (−0.1262) and high positive DG (1.0431). The S and ARA breeds ranked second and third, respectively (HT = 0.6422 and HT = 0.3975, respectively). The S breed displayed a positive HS (0.4664) and positive DG (0.1758). Conversely, the ARA breed displayed a negative HS (−0.4014) and positive DG (0.7989). Breeds such as CNO, GWL, LTC, CNW, C, GWR, and GWY exhibited lower positive HT values ranging from 0.0846 (GWY) to 0.2890 (CNO). Among the breed populations, CNW had the highest HS value (1.0306) and a high negative DG value (−0.8588), while C had a negative HS value (−0.5416) and a high positive DG value (0.7095). Interestingly, the HB and HYD breeds displayed the highest DG values (1.8318 and 3.721, respectively) with profoundly negative HS values (−1.781 for HB and −3.6835 for HYD). In contrast, HIL12, HIL1, HIL5, and HIL2 exhibited negative HT values, with corresponding negative DG values.

#### 3.2.2. Allele Diversity

Figure 3B presents the genetic contribution of each breed based on allelic diversity (AT), which is further divided into intra-breed allelic richness (AS) and inter-breed allelic diversity (DA) (Supplementary Table S2). The CNW breed population ranked the highest in its contribution to AT (0.5907), followed by the HIL12, GWL, GWR, S, CNO, HIL2, and HIL5 breeds (ranging from 0.2296 to 0.5507). These breeds exhibited a similar pattern of positive AS and negative DA values. The ARA and C breeds had negative AT values (−0.0638 and −0.2816, respectively) accompanied by negative AS values (−0.2917 and −0.6327, respectively) and positive DA values (0.2279 and 0.3510, respectively). The HB and HYD breeds displayed significantly negative AT values (−0.8736 and −1.3973, respectively), with the most pronounced negative AS values (−2.3404 and −4.5959, respectively) and the highest positive DA values (1.4668 and 3.1986, respectively).

## 4. Discussion

The conservation of genetic diversity in livestock is critical to ensure the sustainability of the livestock industry, particularly to enhance breeding efficiency, maintain adaptability in rearing, and bolster disease resistance. This underscores the importance of implementing targeted conservation programmes designed to safeguard genetic diversity, as emphasised in several studies [29,30]. Effective conservation programmes require a thorough assessment of population genetics, which offers foundational insights into genetic variability and informs strategic conservation measures [31]. In this study, genetic diversity across breeds was assessed using PCA, admixture analysis, phylogenetic tree construction, and genetic diversity analyses. Conservation priorities were identified based on gene and allelic diversities. Population structure and diversity analyses revealed clustering among Korean native chicken breeds and locally adapted breeds, with HB and HYD exhibiting genetic divergence from other groups. Korean native and locally adapted breeds demonstrated greater genetic diversity compared to commercial breeds, whereas HB and HYD displayed lower levels of genetic diversity. Conservation priorities, based on HT and AT, were identified as SUL and CNW, respectively. These findings highlight the genetic diversity and population structure of Korean native and locally adapted chicken breeds and emphasise the importance of targeted efforts for their conservation.

The study findings reveal that chicken breeds conserved on private farms in Korea (HB, HIL, HYD, LTC) are genetically distinct from other Korean native and locally adapted breeds (ARA, C, CNO, CNW, GWL, GWR, GWY, S). Specifically, the HYD, HIL, and HB breeds exhibited clear genetic differentiation, which can be attributed to the deliberate selective breeding undertaken by farmers to preserve breed-specific traits. The LTC breed was genetically similar to Korean native breeds, such as CNO, CNW, and GWL, likely due to breeding strategies aimed at enhancing long-tail traits and historical breeding practices [32]. The HIL breed, although phenotypically similar to Korean native breeds, sharing traits such as lighter body weight, was identified as a genetically distinct population [33]. Additionally, admixture and phylogenetic tree analyses indicated the genetic similarity of ARA and SUL, which aligns with the fact that the Gyeongbuk Araucana is a hybrid breed developed through crossbreeding between the Golden Duckwing Araucana and SUL breeds [34].

There are various methods to maintain genetic diversity in small populations. Of these, the periodic replacement of individuals within a population is one of the critical factors contributing to the preservation of genetic diversity. Previous studies [35,36] have highlighted the critical role of maintaining accurate pedigree records and employing systematic mating strategies in preserving genetic diversity within small populations. The findings of this study support these observations, as the LTC breed, consisting of a small population of approximately 200 individuals, exhibited positive Fii and Si values, indicating a high level of genetic diversity (Table 2). These outcomes are likely attributed to the farm’s breeding strategy, particularly the planned mating system that involves the regular replacement of male individuals. Forming multiple groups of female LTCs and periodically introducing new male individuals effectively prevents the restriction of the LTC gene pool.

Selection based on non-economic traits in small populations contributes to maintaining genetic diversity by exerting weaker selection pressures. Traits that include feather colour, horn presence, or skin pigmentation do not directly impact critical functions such as survival, productivity, or reproduction but have significant roles in defining the external and non-functional characteristics of individuals. These traits are predominantly governed by a few major genes, such as the melanocortin-1 receptor and tyrosinase, which are less influenced by intense selection, allowing broader genetic variation to persist and contribute to genetic diversity [37,38,39]. For example, the HIL breed comprises 15 strains, including black, white, yellowish, and greyish varieties, and is selected primarily based on feather colour, which is visually distinguishable and regulated by key genes. Despite being a small population, the HIL breed maintains a higher genetic diversity compared to other privately farmed breeds [40]. This is largely due to the low selection pressure on non-economic traits, which allows native breeds to retain higher genetic diversity than locally adapted breeds, often imported for their productivity as commercial breeds. This provides a crucial genetic reservoir that can support the livestock industry in responding to emerging diseases and evolving commercial demands.

The patterns observed in LD and Ne analyses provide support for the genetic diversity results. Breeds with higher levels of inbreeding, as indicated by F_HOM_ and Ho/He values, correspondingly exhibit higher LD and smaller Ne values. For instance, the HYD and HB breed populations, which had the highest F_HOM_ and lowest Ho and He values, also displayed the largest LD values across all distances. This suggests the limited genetic recombination and strong inbreeding within this population. Conversely, breeds with higher heterozygosity and MAF values, such as CNW and GWR, display more pronounced LD decay and larger Ne values. The CNW breed, for example, displayed relatively high Ho, He, and MAF values, which aligns with its low LD values and faster LD decay, indicating frequent recombination events and a larger effective population size. Breeds such as C exhibit low genetic diversity metrics but maintain the lowest LD and highest Ne values. This pattern can likely be attributed to its origins in a large founding population, followed by intense selective breeding. While initial diversity and recombination maintained a low LD, intensive selection for productivity reduced genetic variation in targeted regions, which is a pattern commonly observed in other livestock species [41,42].

A tailored conservation programme that integrates the genetic diversity status and specific rearing objectives of chicken populations is crucial for their sustainable management. However, the constraints of limited resources demand the strategic prioritisation of breeds to maximise impact and ensure long-term viability. This study evaluated conservation priorities by examining the contributions of subpopulations to AT and HT. The S and SUL breeds displayed the highest contribution to HT, with S demonstrating well-preserved intra-population diversity (HS = 0.4664, HT = 0.6422). In contrast, the SUL breed, despite its negative intra-population diversity (HS = −0.1262), showed significant inter-population differentiation (DG = 1.0431) and unique genetic characteristics. Locally adapted breeds, which consistently display higher DG values indicative of their greater genetic divergence from other breeds, contribute more substantially to HT than native breeds. This highlights their critical role in sustaining genetic diversity within the AGRRC and underscores the importance of prioritising these breeds in targeted conservation strategies.

The analysis of conservation priorities based on HT contributions is mainly influenced by genetic distance, highlighting the importance of incorporating AT into the prioritisation process. While HT represents short-term genetic diversity within populations, AT focuses on long-term evolutionary potential and the preservation of rare alleles [18,22]. Notably, conservation rankings based on AT contributions differ from those derived from HT, with Korean native breeds, such as CNW, HIL12, GWL, and GWR, ranking higher for AT, while most locally adapted breeds, except S, rank lower. This highlights the value of a balanced approach to conservation that accounts for both immediate genetic diversity and long-term evolutionary goals.

The S, CNW, GWL, and CNO breeds are critical in maintaining genetic diversity and should be prioritised for conservation within the AGRRC. This study utilised a straightforward ranking method that integrates HT and AT, which represent complementary metrics reflecting short-term genetic health and long-term adaptive potential, respectively. By assigning equal weight to both metrics, the combined ranking provides an overview of the conservation priorities for each breed. The S breed population achieved the highest overall rank (score = 7), followed by CNW and GWL (score = 8) and CNO (score = 10). In contrast, the HYD breed population had a low rank (score = 26), with HB and HIL1 both ranked at 23. While this simple ranking method effectively summarises the overall priority of each breed, it may not fully capture the distinct roles of HT and AT. Nevertheless, the results underscore the importance of S, CNW, GWL, and CNO as key breeds for maintaining genetic diversity and highlight their priority for conservation within the AGRRC. However, combining HT and AT rankings in this way may oversimplify the balance between short- and long-term objectives, as these metrics measure distinct aspects of genetic diversity. An alternative approach could involve assigning different weights to HT and AT based on their relative importance in specific conservation contexts. Additionally, multi-criteria decision analysis should be considered as a tool to evaluate these metrics independently, enabling a more complex prioritisation framework [43]. By employing such strategies, conservation plans can better balance immediate genetic health with long-term adaptive potential, supporting more comprehensive and effective decision-making. Moreover, while HYD, HB, and HIL breeds rank lower in conservation priority based on their combined rank, the genetic uniqueness of these species was demonstrated through population genetic analyses. This highlights the need for tailored conservation programmes and assessment methods that address the specific characteristics and genetic contributions of unique breeds, ensuring that their preservation is not overlooked.

Although the limitations associated with the ranking method have been acknowledged, considering the additional inherent limitations of this study is important. Due to budget and human resource constraints, the present study did not include all breeds conserved by the government. This study represents an initial step toward establishing conservation strategies for government-managed breeds. Further studies should include a more comprehensive range of breeds. Moreover, the current dataset provides a foundation for subsequent research, including investigations on the dynamics of genetic diversity in response to conservation strategies.

## 5. Conclusions

Monitoring the genetic diversity of chicken populations is a prerequisite for developing efficient conservation strategies. The present study assessed the population structure and genetic diversity of 16 Korean chicken breeds managed by a governmental institute and identified conservation priorities based on genetic diversity. These findings highlight that the S, CNW, and GWL breeds exhibited the highest conservation priority because of their significant contributions to overall genetic diversity. These results provide valuable insights for researchers and policymakers in animal genetic conservation, helping prioritise conservation efforts under limited financial and human resources. However, only basic genetic diversity indices were assessed, and the focus was strictly targeted toward government-managed breeds. Future research should aim to overcome these limitations by expanding the scope of this study to include a more comprehensive range of government-managed breeds and investigating the dynamics of genetic diversity in response to conservation strategies.

## Figures and Tables

**Table 1:** Characteristics and management of Korean native and adapted chicken breeds.

Breed Name	Acronym	N	FAO Class ^1^	Origin Source
Gyeongbuk Araucana	ARA	20	Native	Government
Rhode Island	C	20	Adapted	Government
Chungnam Korean Ogye	CNO	20	Native	Government
Chungnam white Korean native	CNW	20	Native	Government
Gangwon black Korean native	GWL	20	Native	Government
Gangwon reddish Korean native	GWR	19	Native	Government
Gangwon yellowish Korean native	GWY	19	Native	Government
Hwangbong-dak	HB	18	Native	Private
Hyuin black	HIL-1	20	Native	Private
Hyuin white	HIL-12	19	Native	Private
Hyuin yellow	HIL-2	20	Native	Private
Hyuin grey	HIL-5	20	Native	Private
Hoeungseong-yakdak	HYD	20	Native	Private
Long-tail chicken	LTC	20	Native	Private
Cornish	S	20	Adapted	Government
White Leghorn	SUL	20	Adapted	University

^1^ FAO classification.

**Table 2:** Genetic diversity metrics of Korean native and locally adapted chicken breeds.

Breeds	H_O_	H_E_	MAF	F_HOM_	Fii	Si
ARA	0.2421	0.2262	0.1685	0.3071	−0.0611	−0.0231
C	0.2231	0.2181	0.1640	0.2670	−0.0217	−0.0077
CNO	0.2834	0.2723	0.2049	0.2072	−0.0402	−0.0140
CNW	0.3293	0.3078	0.2334	0.1270	−0.0646	−0.0247
GWL	0.3056	0.2904	0.2184	0.1936	−0.0398	−0.0177
GWR	0.3155	0.2970	0.2247	0.1677	−0.0590	−0.0219
GWY	0.3013	0.2794	0.2117	0.1925	−0.0730	−0.0276
HB	0.1387	0.1353	0.1012	0.5872	−0.0245	−0.0084
HIL-1	0.2867	0.2668	0.2010	0.2145	−0.0700	−0.0254
HIL-12	0.3167	0.3033	0.2288	0.1683	−0.0396	−0.0153
HIL-2	0.2982	0.2808	0.2116	0.1975	−0.0584	−0.0213
HIL-5	0.3005	0.2796	0.2102	0.1909	−0.0706	−0.0258
HYD	0.0440	0.0388	0.0306	0.8524	−0.1264	−0.0526
LTC	0.2575	0.2665	0.2002	0.2910	0.0230	0.0115
S	0.2842	0.2755	0.2078	0.2125	−0.0304	−0.0109
SUL	0.2551	0.2420	0.1813	0.1223	−0.0489	−0.0182

## Data Availability

The data presented in this study are available on request from the corresponding author, as the research project was conducted by a national institution.

## Supplementary Materials

The following supporting information can be downloaded at https://www.mdpi.com/article/10.3390/ani15081084/s1. Table S1: Contribution of individual chicken breeds to total gene diversity (HT), intrapopulation (HS), and interpopulation (DG); Table S2: Contribution of individual chicken breeds to total allelic diversity (AT), intrapopulation (AS), and interpopulation (DA); Figure S1: Cross-validation error plot of admixture analysis.
