# Supplementary material for: Genetic Diversity and Conservation Priority of Korean Chicken Breeds Using Single-Nucleotide Polymorphism Markers
Source: Animals (Basel). 2025 Apr 9;15(8):1084. doi: 10.3390/ani15081084 (PMC12024459; doi:10.3390/ani15081084)
Supplement: Supplementary file 1 [file animals-15-01084-s001.zip › animals-3514292-supplementary.pdf]

Supplementary Table S1. Contribution of individual chicken breeds to total gene diversity (HT), intrapopulation (HS), and interpopulation (DG)

Rank	Breeds	HT	HS	DG
1	SUL	0.9169	-0.1262	1.0431
2	S	0.6422	0.4664	0.1758
3	ARA	0.3975	-0.4014	0.7989
4	CNO	0.2890	0.4077	-0.1188
5	GWL	0.2300	0.7267	-0.4968
6	LTC	0.2042	0.3058	-0.1017
7	CNW	0.1718	1.0306	-0.8588
8	C	0.1679	-0.5416	0.7095
9	GWR	0.1040	0.7966	-0.6926
10	GWY	0.0846	0.5044	-0.4199
11	HB	0.0507	-1.7810	1.8318
12	HYD	0.0375	-3.6835	3.7210
13	HIL12	-0.0991	0.8996	-0.9987
14	HIL1	-0.2008	0.3102	-0.5109
15	HIL5	-0.2127	0.5345	-0.7472
16	HIL2	-0.2262	0.5552	-0.7814

Supplementary Table S2. Contribution of individual chicken breeds to total Allelic diversity (AT), intrapopulation (AS), and interpopulation (DA)

Rank	Breeds	AT	AS	DA
1	CNW	0.5907	1.0857	-0.4949
2	HIL12	0.5506	1.1295	-0.5790
3	GWL	0.5416	0.9904	-0.4489
4	GWR	0.4418	0.8969	-0.4551
5	S	0.3739	0.5538	-0.1799
6	CNO	0.3026	0.5148	-0.2122
7	HIL2	0.2365	0.6889	-0.4524
8	HIL5	0.2296	0.6570	-0.4274
9	LTC	0.2252	0.3989	-0.1737
10	GWY	0.2176	0.5209	-0.3033
11	SUL	0.1999	-0.0263	0.2261
12	HIL1	0.1203	0.4501	-0.3299
13	ARA	-0.0638	-0.2917	0.2279
14	C	-0.2816	-0.6327	0.3510
15	HB	-0.8736	-2.3404	1.4668
16	HYD	-1.3973	-4.5959	3.1986

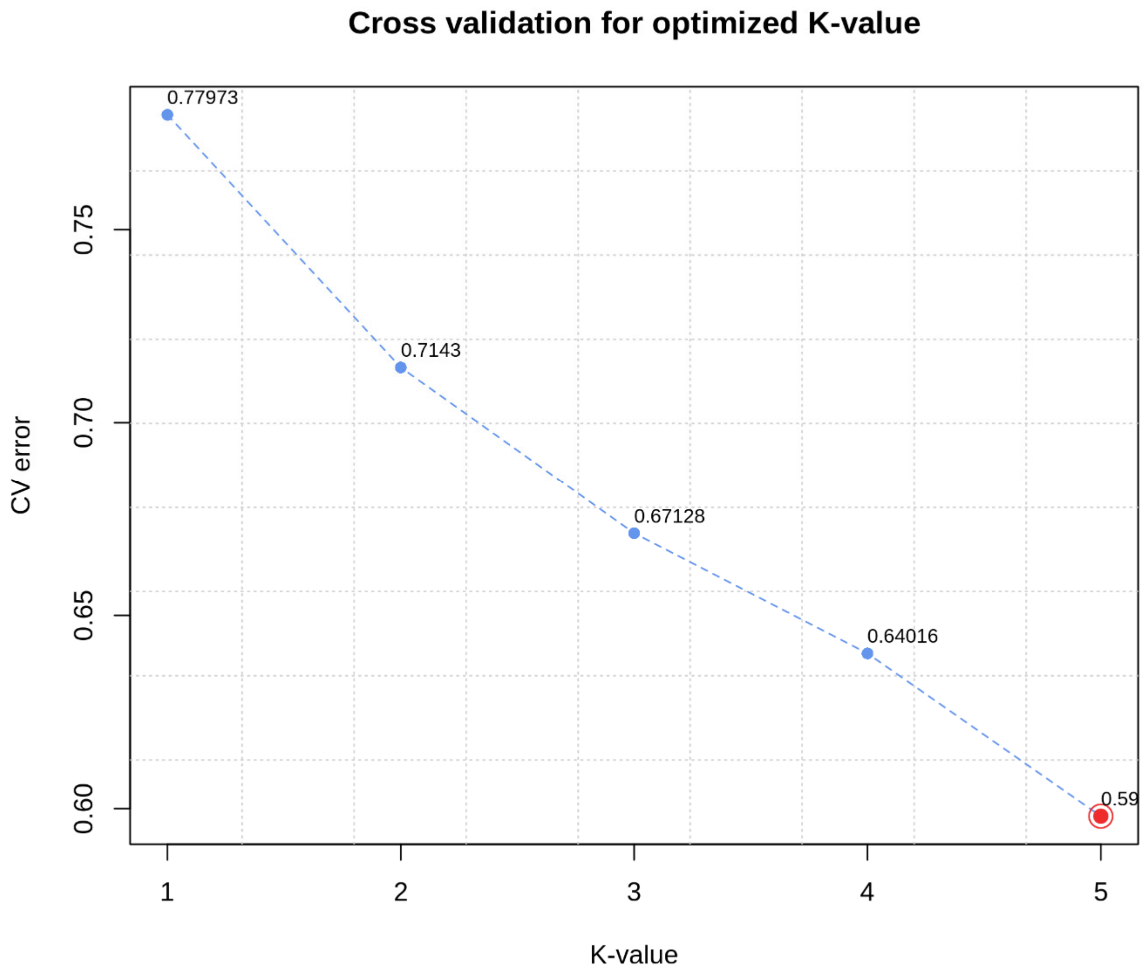

Supplementary Figure S1. Admixture analysis cross-validation error plot.
